# Improving communication and recall of information in paediatric diabetes consultations: a qualitative study of parents’ experiences and views

**DOI:** 10.1186/s12887-015-0388-6

**Published:** 2015-06-10

**Authors:** Julia Lawton, Norman Waugh, Kathryn Noyes, Kathryn Barnard, Jeni Harden, Louise Bath, John Stephen, David Rankin

**Affiliations:** Centre for Population Health Sciences, The Usher Institute of Population Health Sciences and Informatics, University of Edinburgh, Medical School. Teviot Place, Edinburgh, EH8 9AG, UK; Health Sciences, Division of Health Sciences, Warwick Medical School, University of Warwick, Coventry, CV4 7AL, UK; Royal Hospital for Sick Children, Sciennes Road, Edinburgh, EH9 1LF, UK; Human Development and Health, University of Southampton, Southampton General Hospital, Tremona Road, Southampton, SO16 6YD, UK; Child Health Department, Borders General Hospital, Melrose, TD6 9BS, UK

**Keywords:** Type 1 diabetes mellitus, Paediatric consultations, Parents, Qualitative research

## Abstract

**Background:**

Parents of non-adolescent children with type 1 diabetes are responsible for most of their child’s diabetes management tasks. Consultations are used to provide diabetes education, review clinical progress and promote diabetes management tasks. This study explored parents’ experiences of, and views about, their child’s diabetes consultations. The objective was to identify ways in which consultations could be improved to aid communication, understanding and knowledge retention.

**Methods:**

In-depth interviews with 54 parents of children (aged ≤12 years) with type 1 diabetes. Data were analysed using an inductive thematic approach.

**Results:**

Parents’ accounts revealed structural and contextual factors which could hinder effective communication and knowledge acquisition during consultations. Most reported feeling anxious going into consultations and worrying about being reprimanded by health professionals if their child’s glycaemic control had not improved. As a consequence, many parents highlighted problems concentrating and assimilating information during consultations. In extreme cases, worries about being reprimanded led parents to omit or fabricate information when discussing their child’s treatment or even to their cancelling appointments. Many parents described wanting opportunities to speak to health professionals alone because young children could be distracting and/or they did not want to raise distressing issues in front of their child. Parents described the benefits of receiving clinical advice from health professionals familiar with their family circumstances and disliking attending busy clinics and seeing different health professionals on each occasion. Parents also highlighted the benefits of receiving treatment recommendations in a written form after the consultation.

**Discussion and conclusions:**

This study has highlighted unrecognised and undocumented aspects of the consultation which may result in parents leaving uncertain about the main issues discussed and with questions unanswered and support needs unaddressed. Structural and contextual changes to consultations are recommended to improve concentration, knowledge acquisition and retention. These include: sending letters/written summaries after consultations highlighting key decisions, providing opportunities for parents to consult health professionals without their child being present, encouraging parents to ask more questions during consultations, having procedures in place to promote continuity of care and providing parents with consistent and non-contradictory advice.

## Background

Type 1 diabetes usually develops in childhood or adolescence, and its incidence is rising rapidly globally [1]. Optimal blood glucose control in childhood can significantly reduce the risk of complications in adulthood. However, many children experience sub-optimal control [2, 3]. Amongst non-adolescent children, parents assume overarching responsibility for a complex array of diabetes management tasks, including: checking their child’s blood glucose levels, calculating and administering insulin doses, carbohydrate counting, and preventing hypoglycaemia and hyperglycaemia [4].

The consultation is the main method used by healthcare professionals to provide, update and reinforce diabetes education, review clinical and other progress, make decisions about treatments and treatment changes; and, support motivation to undertake diabetes management tasks. However, as studies of consultations involving adult patients have shown, there may be significant disagreement between participants and health professionals about what they consider the main outcomes of diabetes consultations to be and participants may leave their consultations with questions unanswered [5, 6], with detrimental implications for their diabetes (self-) management practices [5, 6]. While most research to date has focused on adult diabetes consultations [5–9], only a minority of studies have explored paediatric consultations and, more specifically, the perspectives of parents and/or their child’s health care providers [10–14]. In keeping with the broader literature on adult consultations, studies of paediatric diabetes consultations have tended to focus on health professionals’ consultation skills and communication styles and how these could be capitalized upon and/or improved to promote parental knowledge, confidence and experience [10, 13, 14]; optimize health care delivery [12, 14]; and, improve the child’s clinical outcomes [12, 14].

To enhance the limited literature on paediatric diabetes consultations, and as part of a broader investigation of why children with type 1 diabetes experience sub-optimal glycaemic control, we explored parents’ experiences of, and views about, participating in their child’s diabetes consultations. Our aims were to identify ways in which paediatric diabetes consultations could be improved from parents’ perspectives to aid communication, understanding and knowledge retention and, hence, to optimize effective use of these consultations.

## Methods

In-depth interviews were undertaken with parents of children ≤12 years as these afforded the flexibility and privacy needed for them to discuss issues they perceived as salient, including those not anticipated at the study’s outset [15, 16]. Data collection and analysis took place concurrently, in line with an inductive approach, enabling issues identified in early interviews to inform the areas explored in later ones [17] Recruitment and interviewing continued until no new findings were identified in new data collected.

### Recruitment and sample

Parents were recruited from four Scottish paediatric diabetes centres. These centres were selected on the basis that they spanned diverse rural and urban catchment areas, and employed between one and four consultants, to enable parents to report potentially diverse experiences of consultations. Consultations were delivered by diabetes specialist nurses and dietitians as well as paediatric diabetes consultants in all four sites.

Parents were recruited by health professionals using an opt-in procedure and purposive sampling was used when arranging interviews to ensure that there was diversity in the final sample in terms of their child’s: age, gender, diabetes duration (at least six months), regimen (multiple daily injections and CSII) and glycaemic control (HbA_1c_) and parents’: education, occupation, employment status (full-time, part-time) and marital status. The final sample comprised 54 parents and included 38 mothers and 16 fathers of 41 children aged ≤12 yrs (see Table 1).

**Table 1:** Demographic characteristics of interview participants and their children

Characteristic	N	%	Mean ± SD & range
Parents (*n* = 54)^a^			
Female (mothers)	38	70.4	
Age - All parents (Years)			40.6 ± 6.1, range 25–51
Mothers Age (Years)			40.0 ± 5.6, range 25–51
Fathers Age (years)			42.1 ± 7.0, range 27–51
Biological parents living together (data from 40 interviews)	28	70.0	
Current employment status			
Full-time	19	35.2	
Part-time	18	33.3	
Full-time carer	7	13.0	
Not working	9	16.7	
In education	1	1.8	
Occupation			
Professional	9	16.7	
Semi-skilled	12	22.2	
Unskilled	17	31.5	
Full-time carer/not working	16	29.6	
Education – (those with degrees)	15	27.8	
Children (*n* = 41)^b^			
Female	17	41.5	
Age – all children			8.4 ± 2.5, range 2–12
Female age at time of interview (Years)			9.0 ± 2.2, range 5–12
Male age at time of interview (Years)			8.0 ± 2.7, range 2–12
Female age at diagnosis (Years)			5.2 ± 2.1, range 3–10
Male age at diagnosis (Years)			3.6 ± 2.3, range 1–8
Diabetes duration – all children (Years since diagnosis)			4.1 ± 2.9, range 1–11
Regimen (at time of interview)			
Basal Bolus	26	63.4	
Mixed-use insulin	2	4.9	
CSII	13	31.7	
HbA_1c_ – all children (IFCC: mmol/mol; NGSP: %)			68 ± 12.3; 8.4 ± 1.1

^a^A total of 40 interviews were conducted. Of these, 24 interviews were with mothers only, 2 with fathers only and 14 were joint interviews with both mothers and fathers

^b^Details of 41 children are provided as one set of parents cared for two children with type 1 diabetes

### Data collection

Interviews were conducted by DR, an experienced qualitative researcher, using a topic guide developed in light of literature reviews, original research questions, preparatory observations of clinics/diabetes consultations, inputs from Advisory Group members (see below), and revised in light of emerging findings. Key areas explored are reported in Table 2. Interviews took place between November 2012 and June 2013 in parents’ own homes, with 14 mother-father dyads choosing joint interviews. Interviews averaged two hours, were digitally recorded (with written consent) and transcribed in full.

**Table 2:** Topic guide questions on parents’ experiences of clinic consultations

• How often do you and your child attend clinic? Who attends the clinic with you? Can you talk me through what usually happens when you attend at clinic?
• How do you feel about the way the clinic is run?
• How do you feel about the information/advice received at clinic?
• How is your child involved in the consultation?
• What other forms of advice/support provided at the clinic would help you?
• Do you have any contact (e.g. phone/email) with any of the clinical care team in between scheduled appointments? When and why would you get in touch?
• For parents who have missed appointments: What are the reasons for this? What might make it easier for them to attend in the future

### Data analysis

JL and DR undertook a thematic analysis of the data using the method of constant comparison [17]. This entailed individual interviews being read through repeatedly before being cross-compared to identify issues and themes which cut across parents’ accounts. In undertaking this comparative analysis, particular attention was paid to differences and similarities in parents’ experiences of, and views about, their child’s diabetes consultations, and the reasons underlying these differences and similarities. All data were reviewed independently before JL and DR met to compare interpretations, discuss discrepant cases and reach agreement on recurrent themes and findings and agree on a coding framework which captured original research questions and emerging findings. Data were coded and retrieved using NVivo, a qualitative software package (QSR International, Doncaster, Australia) and coded datasets were subjected to further analyses.

### Study advisory group

Key findings from the analyses were presented to an Advisory Group comprising health professionals (paediatric diabetes consultants and diabetes specialist nurses), lay representatives (parents of children with type 1 diabetes), policy makers, representatives from JDRF Scotland, NHS Education for Scotland, the National Paediatric and Adolescent Diabetes Co-ordinator; and, academics. The brief of this group was to review the findings in order to make recommendations for clinical practice which were feasible and implementable. This group also advised on the contents of the original interview topic guide.

Ethical approval was provided by the South East Scotland Research Ethics Committee 01, NHS Lothian (12/SS/0071). Below, data are tagged using unique identifiers, with ‘M’ or ‘F’ signifying a child’s mother or father respectively.

## Results

### Styles of communication

In keeping with findings of other studies [12, 13], some parents highlighted difficulties understanding the information imparted during their child’s consultations due to what were seen as ineffective styles of communication, especially amongst diabetes consultants. Indeed, while some consultants were praised for their clear delivery of information, several parents pointed to their excessive use of complex medical terminology which they could find alienating and bewildering. This included 32M who described how “when he [consultant] started going to their whole scientific element of it, I was completely lost” (32M) and 34M who reported how, “he [consultant] speaks about things and graphs and charts and things… I just feel like he’s talking to another doctor or consultant… he doesn’t really speak in my terms” (34M).

In addition to this cross-cutting finding, which is already established in the literature [12, 13, 18], analysis of the interviews revealed structural and contextual factors which could also hinder effective communication and knowledge acquisition during consultations. As we will show, these structural and contextual factors could result in parents leaving consultations confused and uncertain about the issues which had been discussed and treatment decisions made. Parents could also leave with questions unanswered and their own support needs unaddressed. Below, we report these findings in more detail together with parents’ recommendations for how the structural and contextual features of consultations and clinics could be improved to address the problems they encountered. To do this, we begin by exploring the emotional impact of attending consultations on parents and how this could affect their ability to concentrate and, hence, retain and recall information.

### Anxiety and poor recall of information

Virtually all parents described the experience of attending their child’s diabetes consultations as stressful and anxiety provoking, with one mother likening this experience “to going into an exam or something… where you feel sick” (06M). As parents suggested, their anxieties going into their child’s consultations arose from their concerns that, despite their efforts to manage their child’s diabetes, their child’s blood glucose readings and/or HbA_1c_ result would reveal a lack of improvement or even deterioration in their glycaemic control. Hence, parents described worrying about being reprimanded and judged by health professionals: “you feel like you’re instantly going to get told off” (02M); “you think you’re going to be ripped to shreds” (35M) with some describing having been rebuked in the past. Parents also described how their anxiety and upset could be heightened if their concerns were realised and their child’s blood glucose control had not improved. In such situations, parents reported experiencing both imposed and self-imposed feelings of failure and of having let their child down, wherein, “you feel like the worst mother that possibly lived” (23M), and “you feel like you’re failing and they [health professionals] sort of point that out to you, and so you do get anxious” (02M).

As a consequence of their anxieties and emotional reactions, parents highlighted how their concentration levels and, hence, ability to assimilate information during their child’s consultations could be severely compromised. This included 24F who reported poor concentration in his child’s consultations because, “of the various stress levels once they’ve given the blood results”; and, in a more extreme case, 21M, who likened her child’s most recent consultation, where an unexpectedly high HbA_1c_ result was disclosed, to “being in a car crash I suppose… you think you remember what was discussed at the time, but you very, very quickly can’t remember.” Indeed, while parents were keen to praise the clinical advice and input from health professionals, most described struggling to recall all the information these professionals imparted to them at the time:

“The advice is normally good, yeah, normally really good. But, like I say, sometimes later on in the day I’m like that, ummpph, what did he say, what am I meant to be doing?” (05M).

#### Strategies for addressing poor recall of information during consultations

Due to their difficulties assimilating and recalling information, parents highlighted the benefits of receiving clinical advice and recommendations in a written form after the consultation, or indicated a need for information to be given to them in this way: “it would be quite helpful, you know, if they sort of wrote down, you know, the key points that we’re meant to be covering for the next visit” (12F). Others described attempting to overcome their difficulties by “always try[ing] to take some notes” (24M) or bringing along a friend or relative, “because if I’ve forgotten something, then she’ll [mother] remember something” (31M).

In some of the sites included in this study, letters were sent out to parents after the consultations summarising the key action points and treatment changes which had been discussed. This method of dissemination was praised by parents not only because it aided recall of information but also because, as 02F suggested, “if you have it in writing afterwards, you know, it’s typed and you can digest it in your front room” (02F). Parents also described how written summaries enabled them to cascade information to others involved in their child’s care; in 12Fs case, his ex-partner:

“they’re a confirmation of the conversation you’ve had and the settings you’ve changed, and I usually hand them to her Mum so she knows what’s happened, cos she’s not at the meetings, she can see what’s going on.” (12F)

### Non-disclosure in consultations and disengagement

As well as hampering their concentration in consultations, parents’ worries about being judged and reprimanded by health professionals led some to omit or fabricate information about their child’s treatment and care. This included 01M’s practice of, on occasions, substituting high/low blood glucose results in her child’s diary with “a fake reading” after “being told off by the consultant and everybody else, the dietitian and everybody else” after a reading of 2.7 mmol/l was noted in her child’s blood glucose records. Other acts of non-disclosure were also reported by some parents, such as by 34M, to minimize the perceived risk of being chastised:

“Sometimes you don’t want to tell them that you’ve not kept the diary and things like that, you know, you’re going to get into trouble… ‘by the way I’ve never followed your advice, she’s been having her insulin after [her meal]’, I wouldn’t tell them, no.” (34M)

In extreme cases, parents described not attending appointments to avoid being reprimanded. This practice tended to occur at crisis points in parents’ own lives which compounded their difficulties managing their child’s diabetes and left them feeling more vulnerable and unable to cope with what they saw as criticisms from health professionals. Such crisis points included the death of a close relative, or, in 28M’s case, separation from her husband, which caused her to be “away in another world, it was quite a traumatic time” and which resulted in her:

“cancelling appointments cause I didn’t want to go to them, I didn’t want to face them, cause I knew his levels weren’t right and I didn’t want to go in, cause you always go into the clinic thinking you’re going to get a row.”

### The child’s presence in the consultation

Parents also described how having their child present in the consultation could hinder communication and disclosure of information as well as affecting their concentration and, hence, ability to assimilate information. In the case of parents of infants and toddlers, poor concentration was described as arising because:

“you’re trying to digest some information and learn from some of the questions you have … but when you’ve got a three year old, who’s running about sticking her fingers in sockets and looking for attention, it’s just another distraction that you don’t need.” (24F)

Parents of young children also described how, as well as being distracted by their child, they often found themselves having to leave consultations early and with questions unanswered because: “you’ve got him in your lug [ear] saying ‘I want to go, want to go’ and then him getting very agitated and frustrated and it’s like ‘I have to go.’” (10M)

Limited attention spans and disruptive behaviour were also described as extending to children of older ages. In addition, parents voiced their worries that, as their child got older, they were able to understand the implications of what was being discussed in their consultations. As a consequence, parents described being reluctant to raise certain issues or ask certain questions in front of their child, such as those relating to their risk of future complications, due to their wish to protect their child from anxiety and distress:

“there are some questions that you want to ask and so you try and speak a bit in code but then you don’t know if she’s listening, you know, you want, especially at the beginning, you know, ‘oh if we keep up with this HbA_1C_ are her eyes going to be affected’ and, you’re going, trying to do that, you know, it’s like [whispering] the side effect, ‘what about her, her fingers and toes and her…’, so it’s very difficult to ask anything.” (17M)

Parents also described feeling unable ask to health professionals for reassurance and support in front of a child who “understands a lot more than people realise” (24F; child aged 6 years) because, as 06M explained, they did not want to worry or distress their child by appearing to be worried, vulnerable and unable to cope:

“he [son, aged 6 years] needs me to be Mummy and be really strong for him and say, ‘right, it’s fine we’re going to make’, and you know, and say, ‘yes, we’ll take all this on board and we’ll make all the changes and we’ll get it down for next time’ and I can’t sit there and go, ‘aaaarrrrggghh’ to the doctor or to the nurse because that’s, you’re not going to be helpful to [son's name] at all.” (06M)

For the above reasons, some parents highlighted a need for their child to be absent from the consultation for at least part of the time or to be offered other opportunities to talk to health professionals on their own:

“they need to consider catering for parents with a, a younger child who has been diagnosed and they need to, perhaps, facilitate more of a, a one-to-one outwith the presence of the child.” (24F)

In some clinics, provisioning had been made available for children in the form of supervised play areas. Having opportunities to spend time alone with health professionals was uniformly welcomed by parents who attended these clinics as these enabled them “to get some things of your chest”, and ask questions without worrying about upsetting their child. While parents of older children also highlighted the benefits of having time alone with health professionals to ask questions and discuss their worries and concerns, such parents also suggested that excluding or removing their child from the consultation would be inappropriate because:

“They’re speaking more to her now and trying to get her to answer the questions as opposed to looking to us as parents as being the ones responsible… they’re getting her to understand that ‘it’s your condition you need to start taking baby steps towards being responsible for it.’” (21F; child aged 10 years)

### Structural and organizational features of clinics

Parents’ accounts also highlighted other structural and organizational aspects of clinics which could impact on their willingness and ability to disclose information during, and make effective use of, their child’s consultation. In general, parents who attended smaller clinics and encountered the same health professionals conveyed more positive consultation experiences. In particular, these parents highlighted the benefits of receiving clinical advice from health professionals who were familiar with their family set up and personal circumstances and, hence, able to tailor advice to their child. Such parents, including 32F, also described being more likely to adhere to health professionals’ recommendations and follow their advice as a consequence of these personalised and tailored approaches:

“Dr [surname] is good, she understands and she knows the family, she’s known us since he was diagnosed and she knows all of the problems that we’ve had, and she tries to make things as easy as she can for us, she helped me when it was carb counting, it just seemed an absolute nightmare, but she encouraged us to stick with it”.

In comparison, parents who attended larger clinics often described feeling that their child was “just a number” (19M), on a “production line” who was seen by a different health professional on occasion, “who doesn’t know anything about him really” (25M). Parents also perceived the health professionals who worked in these clinics as lacking familiarity with their child and as offering generalized clinical advice as a consequence that is “not going to be about [son’s name], it’s going to be generalised about the whole population of children at his age” (32M). Parents also described disliking seeing different professionals on each occasion as they felt they had to “start right from scratch” (17M) and go right through their child’s diabetes history with each new person encountered. Some parents also pointed to occasions when their lack of familiarity with staff had resulted in them not asking for help and support. This included 25M who had felt unable to share her upset with staff after discovering her son was “one outside his target range for a [Hb]A_1c_” for the first time in several years:

“the care has been fantastic, so em I mean I couldn’t fault it except that when you go up to clinic it can be anyone and so it was a doctor I had never met before and a nurse I had never met before and they didn’t know him or us … and when it’s like there didn’t seem any point in saying ‘och I’m really disappointed’ you know.” (25M)

Parents also highlighted how lack of staff continuity could lead to the receipt of different and sometimes contradictory information which could cause them to feel confused and uncertain about which advice to prioritise and follow:

“they all have slightly different takes on what’s happening and what you should be doing … in the sense that you might see one guy who’s particularly interested in injection sites, but the next time we saw somebody else who was interested in blood glucose readings, so you’ll get different advice, whereas had we, had we seen the injection guy again, we’d have gone into injections… And, we were once suggested, cause pasta's slow burning, that we should inject her after her meal, if she’s having pasta… But then the next consultant said, ‘mm, no, I wouldn’t do it like that.’” (26F)

Some parents also shared their worries that clinics were over-stretched and described how this mitigated opportunities to ask questions because of their concerns about the knock on effects on other parents:

“that’s at the back of your mind, when you’re talking to doctors, you think, maybe, I hope I’m not holding up other people. So that’s, that’s the worst of it, really, 'cause you want to ask them and talk about things but then you don’t want to be making them [late], 'cause I think 'cause I’ve waited for quite a while before, so I kind of feel guilty about making other people late.” (16M)

## Discussion

Key findings arising from this study:

- Parents may experience anxiety resulting in poor recall of information during consultations
- Some clinic staff were described as using over-complex language
- Parents highlighted the desirability and benefits of being provided with written summaries after the consultation
- There was a strong feeling amongst some parents that they were being cross-examined and judged; this could lead to non-disclosure of information and even to non-attendance
- Some parents highlighted a need to be freed from the distraction of infants and young children during consultations
- Parents also described a need for private communication with health professionals without their child present
- Many parents perceived pressures on time during consultations which inhibited them from asking questions and raising concerns
- Parents may get different and contradictory advice from different health care staff
- Parents want and appreciate continuity of care and consistent messages from health professionals.
- These is considerable variation in practice amongst and across services

This is one of few qualitative studies to have explored, in-depth, parents’ experiences of, and views about, participating in paediatric diabetes consultations. In keeping with findings from other studies [12–14], and mirroring health professionals’ perceptions of their own training needs [12], some parents described encountering poor styles of communication during their child’s consultation which could hinder their understanding of, and engagement with, the clinical information discussed. While this finding is already recognised in the literature, and has been reported separately [18], this paper has drawn attention to underreported and undocumented aspects of the consultation and the clinic context which may also result in parents leaving uncertain and confused about the main issues discussed and with questions unanswered and support needs unaddressed.

Specifically, and adding to findings reported by Lowes et al [14], we have shown that parents may be very anxious when they attend their child’s consultations. While anxiety may be a common feature of attending any consultation [19], we have shown that it might be heightened in the case of paediatric diabetes consultations because of parents’ concerns that they may be criticised and reprimanded by health professionals if their child’s glycaemic control has not improved and because they may themselves feel that they have failed their child [18] . We have further shown that this anxiety can result in poor concentration (and hence information retention), non-disclosure of information and, in extreme cases, to parents’ disengagement from future consultations. In addition, we have drawn attention to the ways in which having their child present throughout the consultation, lack of staff continuity and parents’ concerns about clinics being busy and staff over-stretched, can inhibit them from asking questions, retaining all the information discussed during the consultation; and, conveying their own needs for support. As such, these findings may offer useful insights into why parents of children with type 1 diabetes tend to have poor knowledge of diabetes-related complications [20]. Specifically, we have shown that parents are reluctant to ask questions about complications and discuss other sensitive issues in front of their child, an observation also made by Young et al. in a study involving parents with children with leukaemia [21]. Our findings may also enhance understandings of why many children with type 1 diabetes experience suboptimal glycaemic control [2, 3], as well as offering potential ways in which paediatric consultations might be adapted and improved.

To foster effective use of paediatric diabetes consultations and aid positive clinical outcomes, our findings suggest that a multi-faceted approach may be needed. Such an approach would need to extend beyond previously unsuccessful attempts to improve the child’s glycaemic control by targeting health professionals’ consultation and communication styles [22, 23]. Specifically, future approaches would need to address the structural and contextual factors identified in this study which, as we have shown, may also hinder communication, concentration and knowledge-retention. To this end, the following recommendations could be considered for use in clinics (if the procedures and practices outlined below are not in place already) and/or in the development of complex interventions which could be the subject of future research and economic evaluation:

- Letters/written summaries could be sent to parents after the consultations highlighting the key decisions and treatment recommendations made. Alternative, time-saving and cost effective methods of disseminating this information could also be considered and/or piloted, such as the dictation of a memo into a parents’ mobile phone at the end of the consultation.
- To aid parents’ concentration and enable them to ask questions and raise concerns without worrying about upsetting their child, opportunities to have the child absent for part of the consultation could be considered, e.g. through provision of crèches or play therapists within the out-patient setting. As it might not always be appropriate, feasible or desirable to remove or exclude a child from a consultation (e.g. when a young infant does not want to be separated from his/her parents, or when a child grows older and their autonomy needs to be promoted [21]), parents might benefit from telephone follow-up to clarify information and ask questions. Parents could also be encouraged to contact their child’s diabetes professionals between consultations.
- Health professionals need to be aware that parents are worried about asking questions, especially when they perceive clinics to be busy. Hence, they should try to reassure parents at the outset of the consultation that, if there is insufficient time for all of the issues they want to raise to be addressed, additional support outwith clinic could be made available to them.
- To encourage parents to ask questions during the consultation, interventions could be considered which take account of our finding that parents can be distracted and lack concentration in the consultation. This could include Applegate et al.’s [24] simple, low cost intervention which involves getting parents to write down all of their questions in advance of paediatric diabetes consultations and which they showed to have had a positive impact on the number of questions which were subsequently asked.
- Given that parents appreciate continuity of care, they could, when appropriate, be given the opportunity to see the same health professionals at each visit. This could increase opportunities for health professionals to ‘invest’ in families, offer consistent (non-contradictory) clinical advice, and create the affinity and rapport parents feel they need to share any difficulties they are encountering with their child’s care. However, it is noteworthy that all of the clinics included in this study did have procedures in place (e.g. pre- and post-clinic meetings to discuss each child, weekly clinical multi-disciplinary team meetings, and the logging of all information discussed in the child’s clinical records) to aid continuity of care. This would suggest that parents would benefit from being made aware such systems are in operation to help alleviate their concerns about needing to provide historical information about their child and their diabetes management practices.
- In clinics where such procedures are not in place already, continuity of care could be achieved by standardizing practice and having clear policies in place which are known to all staff to help ensure they offer consistent and non-contradictory advice. Health professionals could also consider making a clear note of what was discussed during the consultation and what goals were agreed/set so that this information is available for colleagues to refer to and discuss at subsequent consultations.
- Health professionals need to be aware of how anxious and worried parents of children with type 1 diabetes can be when they attend consultations and how this can compromise concentration, and, in some cases, disclosure of information relevant to treatment decision-making. In extreme cases, this can even lead to non-attendance [25, 26]. Hence, health professionals should take care to deliver test results in a careful, sensitive and non-judgemental ways, to help allay anxiety and poor concentration and make effective use of the consultation thereafter. To this end, additional training for health professionals could be considered.

### Strengths and limitations

Given our use of a qualitative design, our study was necessarily small-scale and this may reduce the generalizability of our findings. Another limitation is that we did not observe consultations and, hence, we are unable to compare parents’ accounts with what actually happened. A key strength of this study is our decision to recruit parents from multiple sites and clinics which had different practices and procedures in place. Not only were we able to compare the experiences and views of parents attending different types of clinics (e.g. those with high/low levels of staff continuity) we were also able to demonstrate that interventions recommended by some parents (e.g. post-consultation letters, child free consultations) were found to be helpful and well received by other parents who had been beneficiaries of such interventions. As such, the findings and recommendations made in this paper could be usefully engaged in routine clinical practice as well as informing the development of complex interventions, which can then be evaluated. Future research could include observations of consultations and health professionals could also be interviewed to investigate their consultation experiences, including their views about benefits and challenges of having a child present or absent during the consultation.

## Conclusions

By using an open-ended, exploratory approach, this study has uncovered issues which have not previously been recognised by clinicians or reported in the literature. Specifically, while poor communication and consultation skills have been widely reported, our findings have highlighted contextual and structural factors which can also hinder communication, understanding and knowledge acquisition during paediatric consultations. We have also provided recommendations for how these contextual factors could be addressed to improve future paediatric consultations and in order to inform the development of complex interventions which could then be the subject of future research.
